# Bioinformatics and computational chemistry approaches to explore the mechanism of the anti-depressive effect of ligustilide

**DOI:** 10.1038/s41598-023-32495-7

**Published:** 2023-04-03

**Authors:** Kun Zhang, Chaoguo Zhang, Xiuli Teng, Ke Wang, Mingwei Chen

**Affiliations:** Department of Respiratory and Critical Care Medicine, First Affiliated Hospital of Xi’an Jiaotong University, 277#, Yanta West Road, Xi’an, 710061 Shaanxi People’s Republic of China

**Keywords:** Computational biology and bioinformatics, Data mining, Gene regulatory networks, Virtual drug screening

## Abstract

Depression affects people with multiple adverse outcomes, and the side effects of antidepressants are troubling for depression sufferers. Aromatic drugs have been widely used to relieve symptoms of depression with fewer side effects. Ligustilide (LIG) is the main component of volatile oil in angelica sinensis, exhibiting an excellent anti-depressive effect. However, the mechanisms of the anti-depressive effect of LIG remain unclear. Therefore, this study aimed to explore the mechanisms of LIG exerting an anti-depressive effect. We obtained 12,969 depression-related genes and 204 LIG targets by a network pharmacology approach, which were intersected to get 150 LIG anti-depressive targets. Then, we identified core targets by MCODE, including MAPK3, EGF, MAPK14, CCND1, IL6, CASP3, IL2, MYC, TLR4, AKT1, ESR1, TP53, HIF1A, SRC, STAT3, AR, IL1B, and CREBBP. Functional enrichment analysis of core targets showed a significant association with PI3K/AKT and MAPK signaling pathways. Molecular docking showed strong affinities of LIG with AKT1, MAPK14, and ESR1. Finally, we validated the interactions between these proteins and LIG by molecular dynamics (MD) simulations. In conclusion, this study successfully predicted that LIG exerted an anti-depressive effect through multiple targets, including AKT1, MAPK14, and ESR1, and the pathways of PI3K/AKT and MAPK. The study provides a new strategy to explore the molecular mechanisms of LIG in treating depression.

## Introduction

Depression, a mood condition, is marked by a continuous sense of melancholy and/or an inability to perceive pleasure, as well as functional limitations^1^. Depression has resulted in adverse outcomes worldwide, including disability and loss of life. Moreover, by 2030, depression is projected to be one of the three leading causes of disease burden^2^. Drug, psychosocial, and neuroregulatory approaches are the most common treatment options for depression^3^. However, the side effects and limited efficacy of these treatments contribute to the low remission rate of depressed patients. Therefore, discovering effective adjuvant drugs with anti-depressive effects is of great clinical importance.

Angelica sinensis has medicinal effects on depression and cognitive disorders^4^, and its active ingredients include ligustilide (LIG)^5^, ferulic acid^6^, d-glucose^7^, etc. Among them, LIG is the main component of the volatile oil of angelica sinensis^8^, which has the properties of easy volatility and easy penetration of the blood-brain barrier, which allows LIG to be delivered to the brain through intranasal administration. Therefore, LIG has a high bioavailability in the treatment of brain diseases^9,10^. Indeed, LIG has shown promising pharmacological effects in neurological and psychiatric disorders. For example, LIG attenuates ischemic brain injury by inhibiting NLRP3 inflammasome activation and microglia focalization^11^ and has potent neuroprotective effects against hemorrhagic stroke^12^. Also, studies have found that LIG can improve cognitive impairment in rats with vascular dementia^5^ and mice with Alzheimer's disease^13^. In addition, the researchers found that Z-LIG, an isomer of LIG, exerts antidepressant-like effects in a rat model of depression^14^. However, the mechanism of the anti-depressive effect of LIG remains unclear.

**Figure 1:**
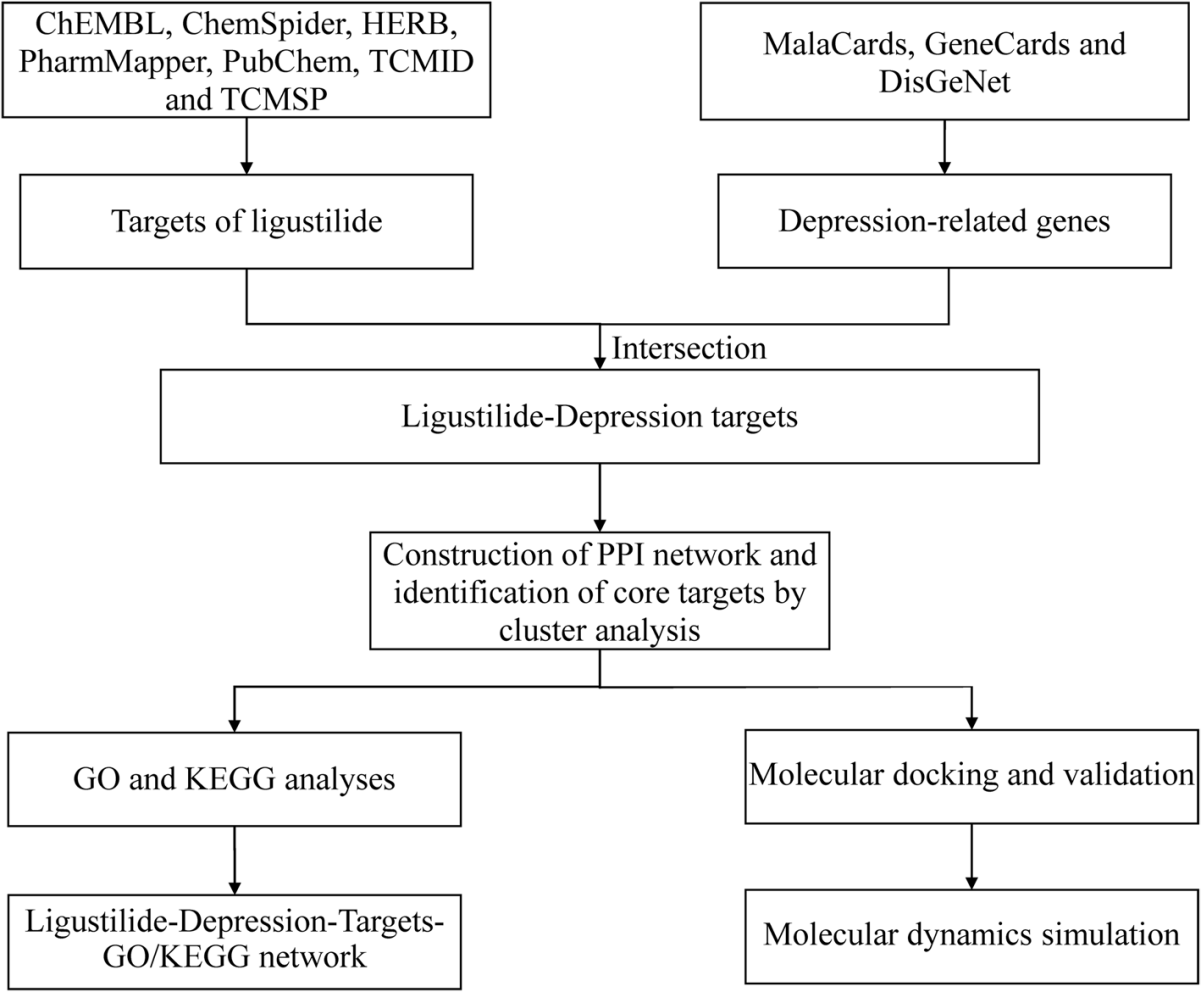
The flow chart for this research. *PPI* protein–protein interaction, *GO* Gene Ontology, *KEGG* Kyoto Encyclopedia of Genes and Genomes.

Therefore, this study aimed to explore LIG's potential pharmacological targets and mechanisms for treating depression through network pharmacology and molecular simulations. Firstly, we collected the related genes of depression and possible targets of LIG, and their intersection was screened out. Then we screened the core targets used for molecular docking with LIG from the intersecting genes. Next, we used molecular dynamics (MD) simulations to assess the protein-ligand interactions and the stability of the complexes. In addition, we performed Gene Ontology (GO) and Kyoto Encyclopedia of Genes and Genomes (KEGG) analyses of the intersecting genes to explore the biological functions and signaling pathways involved in the anti-depressive effect of LIG. The flow chart for this research is shown in Fig. 1.

## Methods

### Collection of depression-related genes

We collected depression-related genes from the MalaCards^15^, GeneCards^16^, and DisGeNet^17^ databases and plotted the Venn diagram using the Venn R package in the R language. The websites of the databases are provided in Table S1.

### Collection of LIG targets

We collected the possible targets of LIG from ChEMBL^18,19^, ChemSpider^20^, HERB^21^, PharmMapper^22^, PubChem^23^, TCMID^24^, and TCMSP^25^ databases, and these databases are commonly used in network pharmacology^26–28^. The websites of the databases are provided in Table S1. We plotted the Venn diagram using the Venn R package in the R language.

### Construction of LIG-depression target network and identification of core targets

Firstly, we compared the depression-related genes with the genes corresponding to the LIG targets to obtain the intersecting genes. We then used the intersecting genes to get the protein-protein interaction (PPI) network (high confidence) in the STRING database (version 11.5)^29^. Then, we used the MCODE APP (default parameters) in Cytoscape software (version 3.7.2)^30^ to perform a cluster analysis of the PPI network, and the targets with the highest clustering score were the core targets.

### GO and KEGG analyses

The core targets obtained above were analyzed for GO^31^ and KEGG^32–34^ by the R package "ClusterProfiler" (qvalue < 0.05). The enrichment analysis results were presented in the form of bar graphs. In addition, the LIG-target-GO function-KEGG pathway-depression-related gene network was constructed using Cytoscape software (version 3.7.2).

### Molecular docking 

We performed bulk molecular docking of LIG and core targets to assess which targets LIG can primarily bind to. The molecular structures of LIG were downloaded from the PubChem database^35^. The protein structures of the core targets were obtained from the PDB database^36^. The PDB IDs of AKT1, MAPK14, and ESR1 are 7nh5^37,38^, 6sfo^39^, and 7msa^40,41^, respectively. We used ChemBio3D Ultra 2014 wizard software to add hydrogen atoms to the LIG and minimize the energy. We used AutoDockTools-1.5.7 for the receptors to remove water molecules, add hydrogen atoms, and generate the coordinate file and for the ligands to perform root detection, select torsions, and generate the coordinate file^42^. The grid boxes included the entire protein structure, and the size and coordinates of the grid boxes and the docking parameters are shown in Table S2. The Root Mean Square Deviation (RMSD) cluster analysis was performed using ligand atoms, which used an RMSD-tolerance of 2.0 Å. Molecular docking was performed using the Autodock Vina program^43^. PyMOL (https://pymol.org/ep, Version 2.5.2, Educational-Use-Only) was used to visualize and show the hydrogen bonds between the receptor residues and the ligands^44^.

### Molecular docking validation

To verify the reliability of the molecular docking results, we used a redocking method to verify the molecular docking method and parameters. The native ligands and receptors were separated from the co-crystal structure and processed for molecular docking as described above. The "align" command in PyMOL was used to calculate the RMSD of the ligand conformation. The redocking protocol was considered valid at RMSD < 2 Å^45^.

### MD simulations

We performed 20 ns MD simulations of protein-ligand complexes obtained by molecular docking using GROMACS software (version 2020.6-MODIFIED) on the Linux operating system^46^. The protein topologies were generated using the AMBER99SB-ILDN force field. GAFF force fields and parameters for the ligands were created using AmberTools and ACPYPE, and the AM1-bcc charges of the ligands were calculated using the antechamber program^47^. The TIP 3-point solvent model was used to dissolve each system and then neutralize the charge with appropriate amounts of Na^+^ and Cl^−^. The steepest descent minimization algorithm was used to minimize the system energy, which stopped minimization at > 50,000 steps and a maximum force of < 10.0 kJ/mol. We performed the NVT and NPT equilibration for each complex system (1 bar pressure and 300 K temperature)^48^. The long-range electrostatics were treated using the Particle Mesh Ewald, and the Fourier transform grid spacing was 0.16. During the 20 ns MD simulation, the time step was 2 fs, and the structural coordinates were saved every 10 ps. We evaluated various parameters of MD, such as RMSD, Root Mean Square Fluctuation (RMSF), and hydrogen bonding (H-bond) monitoring reports^49^. In addition, the non-bond interaction energies between proteins and ligands, including short-range electrostatic interactions and van der Waals (Vdw), were also calculated^50^.

## Results 

### Construction of LIG-depression target network and identification of core targets

**Figure 2:**
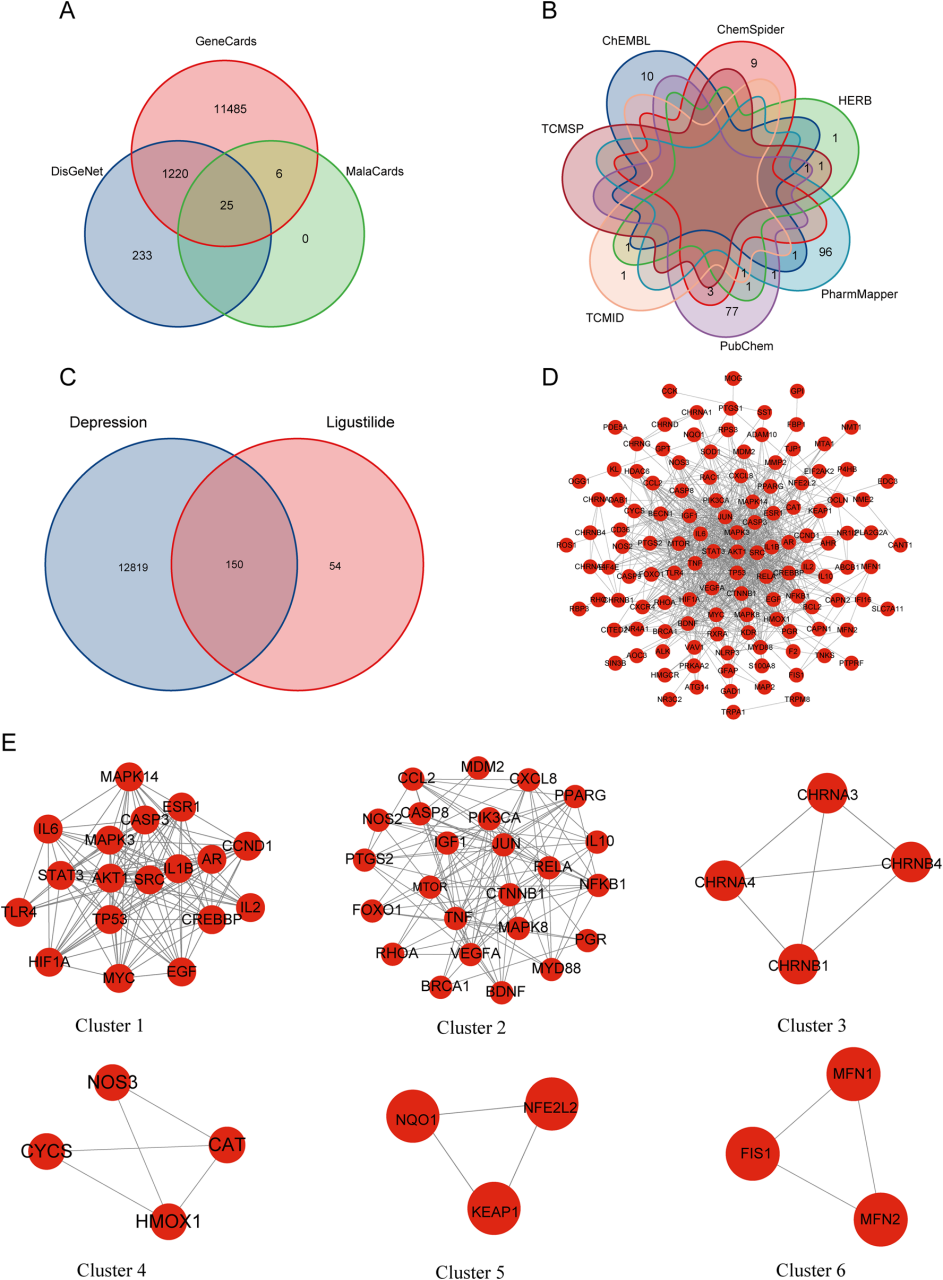
Network pharmacology identifies possible anti-depressive targets for LIG. (**A**) Venn diagram showing genes associated with depression in various disease databases. (**B**) Venn diagram showing the possible targets of LIG in each drug target database. (**C**) Venn diagram shows the 150 common targets between the LIG and depression targets. (**D**) The PPI network of LIG anti-depressive targets. (**E**) The cluster analysis of the PPI network. The 6 clusters contain 18, 24, 4, 4, 3, and 3 nodes, respectively. *LIG* ligustilide, *PPI* protein-protein interaction.

We collected 12,969 depression-related genes (Fig. 2A, Table S3) and 204 possible targets of LIG (Fig. 2B, Table S4). By comparison, we obtained 150 intersecting genes (Fig. 2C, Table S5). Then, we visualized the interactions of intersecting targets by the PPI network (Fig. 2D). The cluster analysis results using MCODE showed that there were 6 clusters in this PPI network (Table 1, Fig. 2E). Cluster 1 had a score of 13.059 and was the core cluster with targets including MAPK3, EGF, MAPK14, CCND1, IL6, CASP3, IL2, MYC, TLR4, AKT1, ESR1, TP53, HIF1A, SRC, STAT3, AR, IL1B and CREBBP (Fig. 2E).

**Table 1:** Clusters in the PPI network are discovered in Cytoscape using MCODE.

Cluster	Score	Nodes	Edges	Node IDs
1	13.059	18	111	MAPK3, EGF, MAPK14, CCND1, IL6, CASP3, IL2, MYC, TLR4, AKT1, ESR1, TP53, HIF1A, SRC, STAT3, AR, IL1B, CREBBP
2	8.957	24	103	BRCA1, CASP8, BDNF, MDM2, RHOA, TNF, CTNNB1, NOS2, IL10, CCL2, NFKB1, CXCL8, FOXO1, MAPK8, IGF1, PGR, PPARG, VEGFA, JUN, RELA, PTGS2, MYD88, PIK3CA, MTOR
3	4	4	6	CHRNA4, CHRNB1, CHRNB4, CHRNA3
4	3.333	4	5	HMOX1, NOS3, CYCS, CAT
5	3	3	3	KEAP1, NFE2L2, NQO1
6	3	3	3	FIS1, MFN2, MFN1

### GO and KEGG analyses

**Figure 3:**
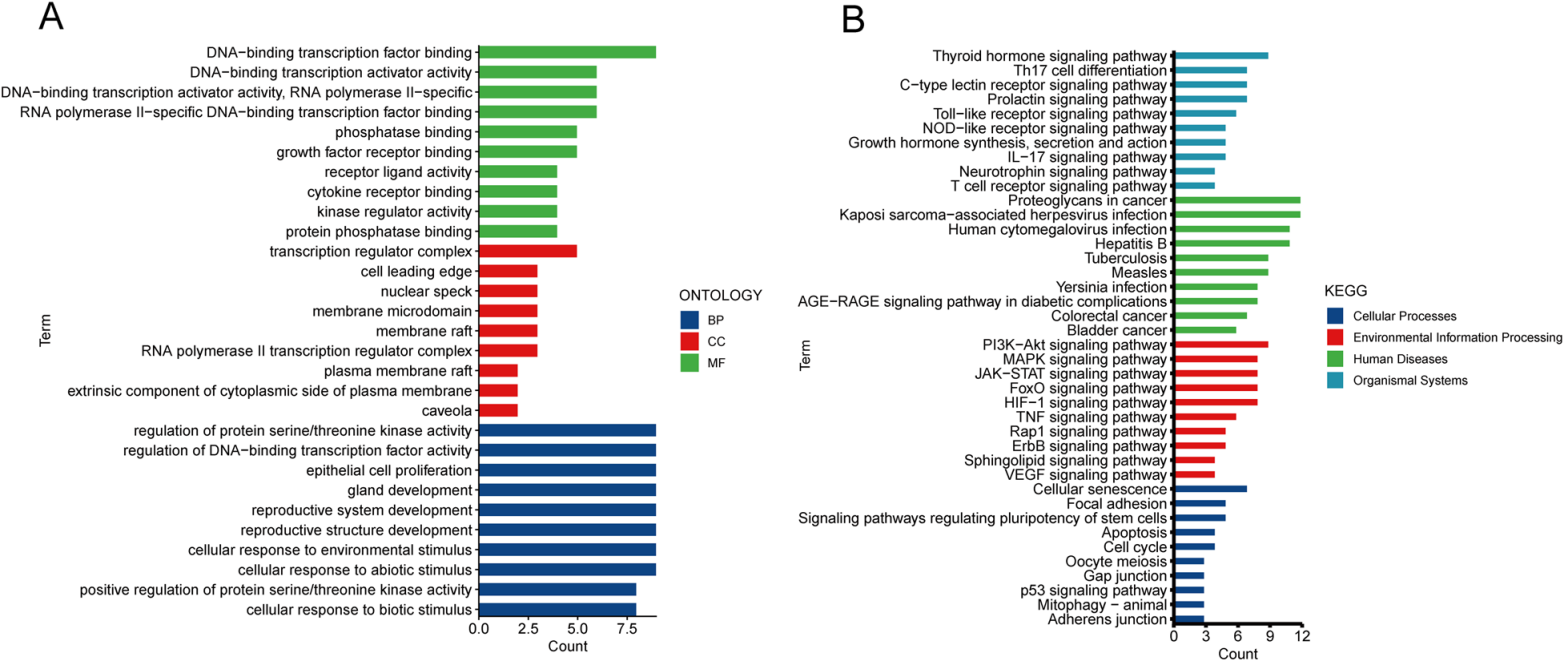
Functional enrichment analysis of core targets. (**A**) Bar plot of GO enrichment analysis (Top 30). (**B**) Bar plot of KEGG^32^ enrichment analysis (Top 41). *GO* Gene Ontology, *KEGG* Kyoto Encyclopedia of Genes and Genomes, *BP* biological process, *CC* cellular component, *MF* molecular function.

We carried out functional enrichment analysis for MAPK3, EGF, MAPK14, CCND1, IL6, CASP3, IL2, MYC, TLR4, AKT1, ESR1, TP53, HIF1A, SRC, STAT3, AR, IL1B, and CREBBP. The results of GO showed that the top-ranked biological process terms were regulation of protein serine/threonine kinase activity, regulation of DNA-binding transcription factor activity, and epithelial cell proliferation, and the top-ranked cellular component terms were transcription regulator complex, cell leading edge, and nuclear speck (Fig. 3A). In addition, the top-ranked molecular functional terms were DNA-binding transcription factor binding, DNA-binding transcription activator activity, and RNA polymerase II-specific (Fig. 3A). KEGG analysis showed that these genes were significantly associated with the thyroid hormone signaling pathway, proteoglycans in cancer, PI3K/AKT signaling pathway, MAPK signaling pathway, and cellular senescence (Fig. 3B).

### Molecular docking and validation

**Figure 4:**
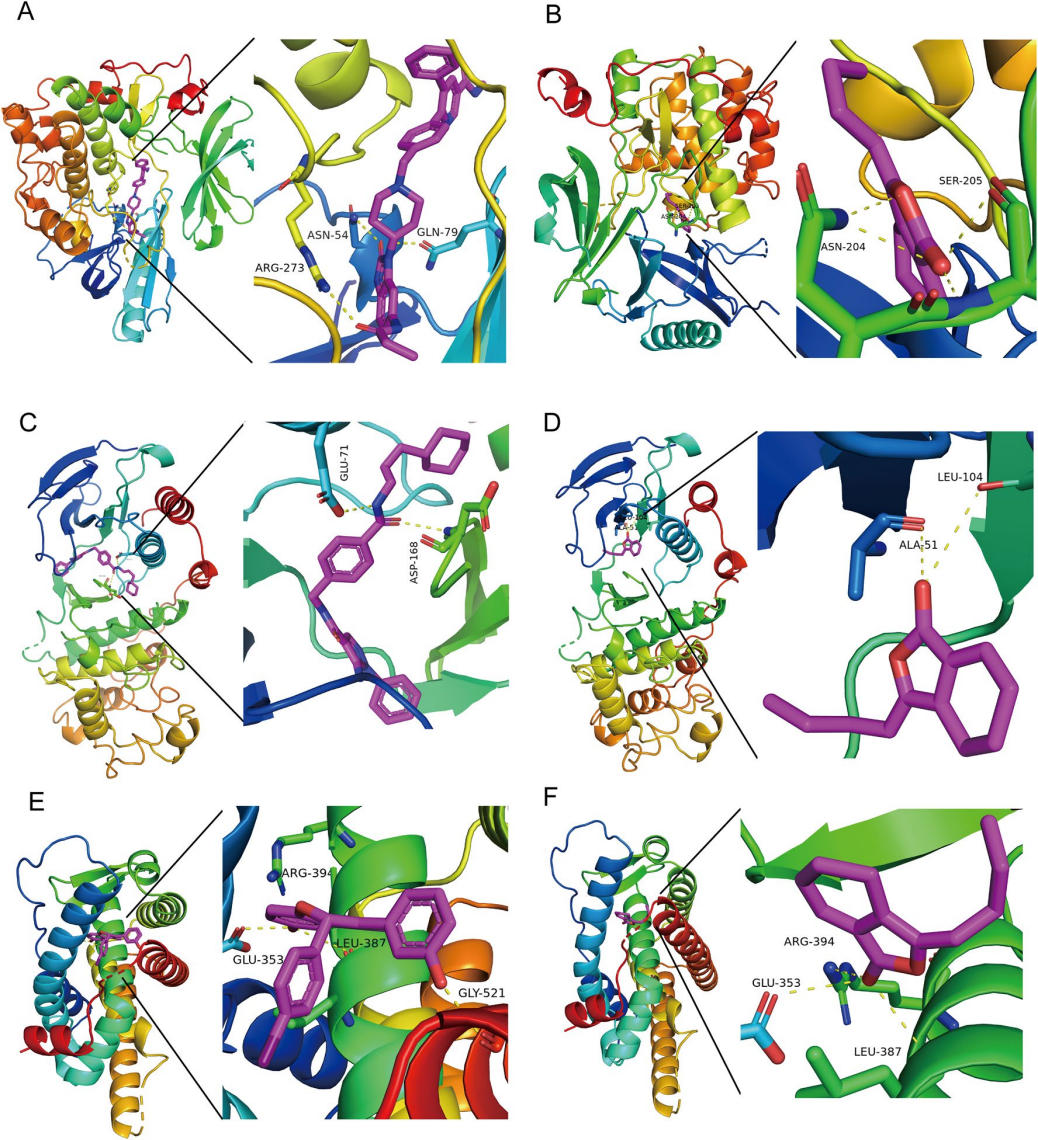
Molecular docking and validation of LIG with AKT1, MAPK14, and ESR1. (**A**) Redocking of the native ligand with AKT1. (**B**) Molecular docking of LIG with AKT1. (**C**) Redocking of the native ligand with MAPK14. (**D**) Molecular docking of LIG with MAPK14. (**E**) Redocking of the native ligand with ESR1. (**F**) Molecular docking of LIG with ESR1. The purple structures represent small molecule ligands, and the yellow dashed lines represent hydrogen bonds.

Molecular docking analysis of LIG and candidate targets showed that LIG had the highest affinity for AKT1, MAPK14, and ESR1 (Table 2). LIG formed 4 hydrogen bonds with amino acid residue ASN-204 and SER-205 of AKT1 (Fig. 4B) with an affinity of − 7.8 kcal/mol (Table 2). LIG formed 2 hydrogen bonds with 2 amino acid residues of MAPK14, including ALA-51 and LEU-104 (Fig. 4D), with an affinity of − 7.3 kcal/mol (Table 2). LIG formed 3 hydrogen bonds with 3 amino acid residues of ESR1, including ARG-394, GLU-353, and LEU-387 (Fig. 4F), with an affinity of − 6.9 kcal/mol (Table 2). In addition, we performed redocking of the native ligands and the receptors to verify the effectiveness of our molecular docking protocol. The results showed that the RMSD value of native ligand UC8 was 1.558 Å after redocking on AKT1. The RMSD value of the native ligand LBE after redocking on MAPK14 was 0.448 Å. The RMSD value of the native ligand ZNM after redocking on ESR1 was 1.196 Å (Table 3). These RMSD values were all less than 2 Å. The binding patterns of native ligands to the receptors are shown in Fig. 4 (Fig. 4A: UC8-AKT1, Fig. 4C: LBE-MAPK14, Fig. 4E: ZNM-ESR1).

**Table 2:** Molecular docking of ligustilide to proteins.

Protein	PDB ID	Affinity (kcal/mol)
AKT1	7nh5	− 7.8
MAPK14	6sfo	− 7.3
ESR1	7msa	− 6.9
CASP3	7seo	− 6.8
AR	2piw	− 6.7
CREBBP	4tqn	− 6.7
MAPK3	6ges	− 6.6
TP53	4agn	− 6.2
TLR4	3fxi	− 6
IL6	1alu	− 5.7
CCND1	5vzu	− 5.6
HIF1A	1h2l	− 5.6
IL2	4nej	− 5.6
IL1B	6y8m	− 5.5
MYC	6g6l	− 5.5
STAT3	6nuq	− 5.3
EGF	1jl9	− 5.1
SRC	1a08	− 4.8

**Table 3:** RMSD values after redocking of native ligands on AKT1, MAPK14 and ESR1.

Protein target	PDB code	RMSD(Å)	Native ligand	Affinity (kcal/mol)
AKT1	7nh5	1.558	UC8	− 13.5
MAPK14	6sfo	0.448	LBE	− 11.6
ESR1	7msa	1.196	ZNM	− 11.7

### MD simulations

#### RMSD and RMSF analyses

**Figure 5:**
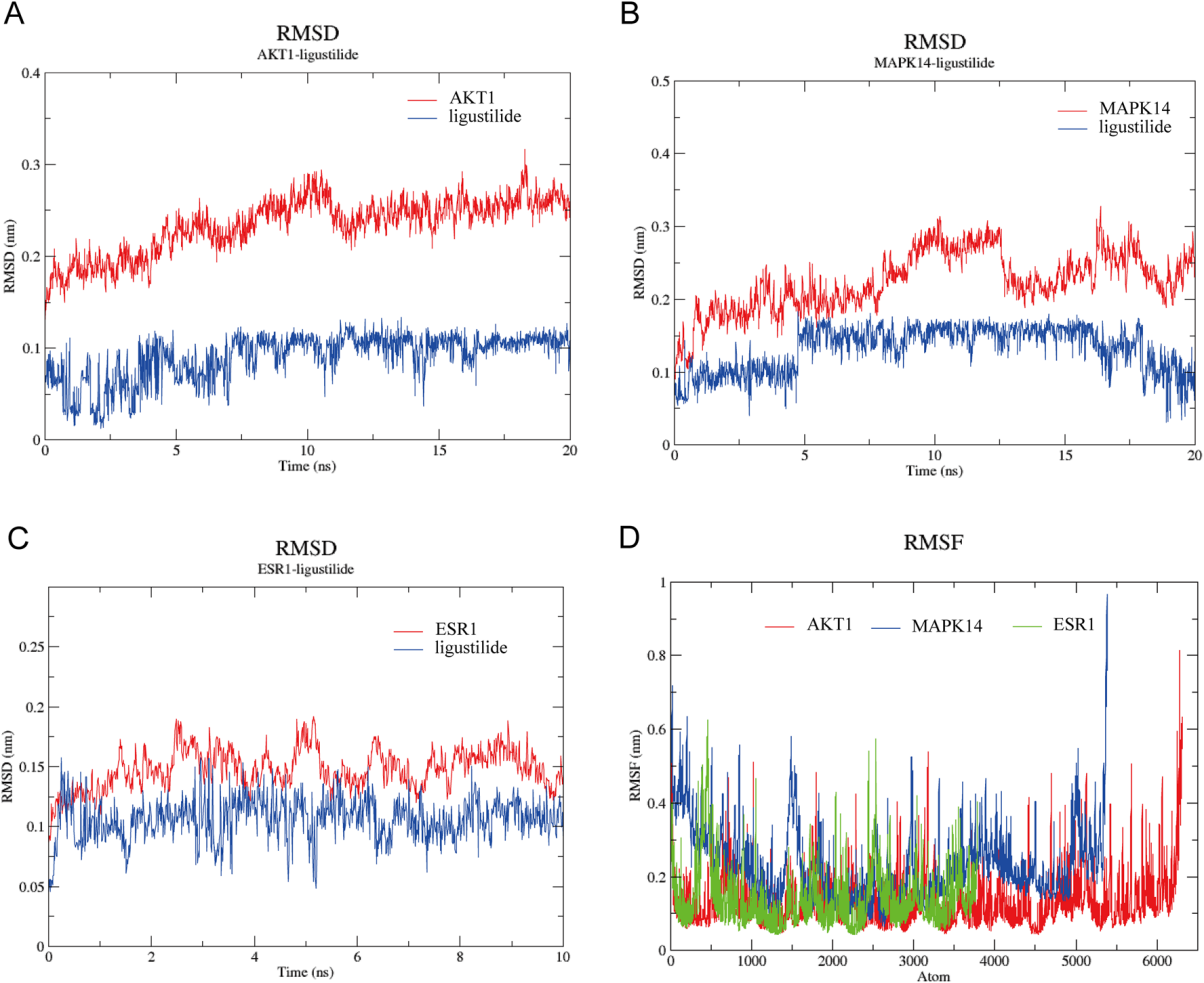
RMSD and RMSF of AKT1-LIG, MAPK14-LIG, and ESR1-LIG complexes. (**A**) RMSD of AKT1-LIG complex. (**B**) RMSD of MAPK14-LIG complex. (**C**) RMSD of ESR1-LIG complex. (**D**) RMSF of AKT1-LIG, MAPK14-LIG, and ESR1-LIG complexes. *RMSD* Root Mean Square Deviation, *RMSF* Root Mean Square Fluctuation, *LIG* ligustilide.

Then, we performed 20 ns MD simulations of the protein-ligand complexes. In the AKT1-LIG complex system, the AKT1 RMSD was in the range of 0.13–0.32 nm with an average RMSD of 0.23 nm, and the LIG RMSD was in the range of 0.01–0.13 nm with an average RMSD of 0.09 nm (Fig. 5A). In the 20 ns MD of MAPK14-LIG, the RMSD of MAPK14 is in the range of 0.08–0.33 nm with an average RMSD of 0.22 nm, and the RMSD of LIG is in the range of 0.03–0.18 nm with an average RMSD of 0.13 nm (Fig. 5B). In the ESR-LIG complex system, the RMSD of ESR1 ranged from 0.09 to 0.19 nm with an average RMSD of 0.15 nm, while the RMSD of LIG ranged from 0.05 to 0.16 nm with an average RMSD of 0.11 nm (Fig. 5C). In addition, we also observed the size of flexibility in specific regions by analyzing the RMSF of the three proteins. The results showed that the AKT1 protein showed the largest RMSF value of 0.82 nm at the 6200–6300 atomic position, while MAPK14 showed a peak RMSF value of 0.97 nm at the 5300–5400 atomic positions, respectively. And the RMSF of ESR1 showed a maximum value at the 440–460 atoms with a maximum value of 0.63 nm (Fig. 5D).

#### H-bond monitoring

**Figure 6:**
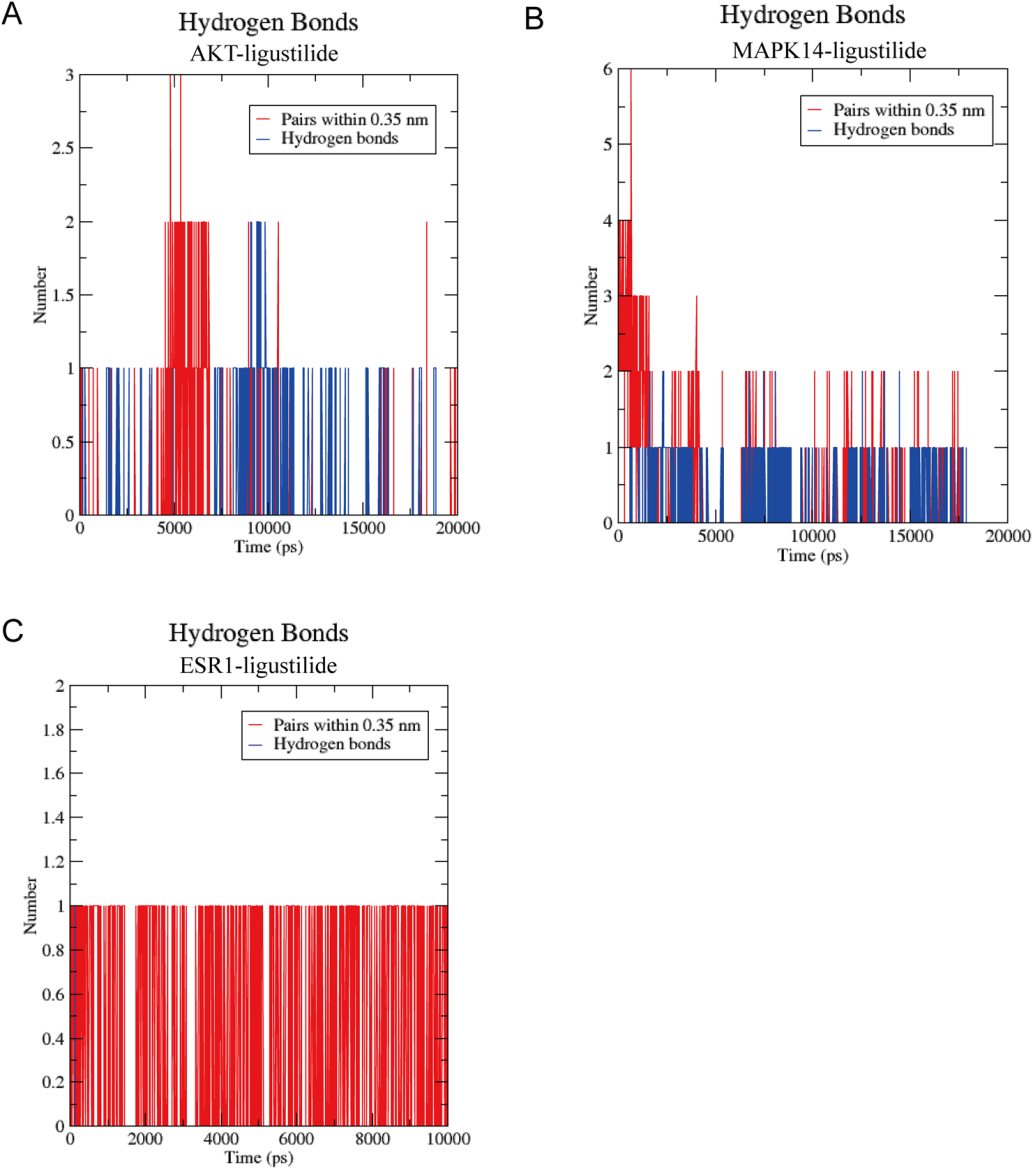
H-bond monitoring reports show temporal changes in hydrogen bonding between protein–ligand complexes. (**A**) AKT1-LIG complex. (**B**) MAPK14-LIG complex. (**C**) ESR1-LIG complex. *LIG* ligustilide.

We monitored the hydrogen bonds formed between proteins and ligands during 20 ns MD simulation. AKT1 and LIG could form 1–2 hydrogen bonds (Fig. 6A). MAPK14 and LIG mainly form 1 hydrogen bond and up to 2 hydrogen bonds within 0–20 ns (Fig. 6B). ESR1 and LIG formed no hydrogen bond in 0–20 ns MD simulation (Fig. 6C).

#### Protein–ligand interaction energy

We also calculated the non-bonding interaction energies, including Coulomb (Coul) and Vdw, between the proteins and ligands to quantify the strength of their interactions. The total interaction energy of AKT1 with LIG was − 131.3 ± 3.8 kJ/mol (Coul: − 17.0 ± 3.3 kJ/mol, Vdw: − 114.3 ± 1.9 kJ/mol). The total interaction energy of MAPK14 with LIG was − 136.6 ± 6.9 kJ/mol (Coul: − 24.6 ± 4.9 kJ/mol, Vdw: − 112.1 ± 4.9 kJ/mol). The total interaction energy of ESR1 with LIG was − 124.4 ± 1.1 kJ/mol (Coul: − 7.1 ± 0.4 kJ/mol, Vdw: − 117.3 ± 1.0 kJ/mol) (Table 4).

**Table 4:** The nonbonded interaction energy between proteins and ligands (kJ/mol).

Protein–ligand complex	Coul	Vdw	Total interaction energy
AKT1-ligustilide	− 17.0 ± 3.3	− 114.3 ± 1.9	− 131.3 ± 3.8
MAPK14-ligustilide	− 24.6 ± 4.9	− 112.1 ± 4.9	− 136.6 ± 6.9
ESR1-ligustilide	− 7.1 ± 0.4	− 117.3 ± 1.0	− 124.4 ± 1.1

*Coul* Coulomb Interactions, *Vdw* Van der Waals Interaction.

## Discussion

Depression, a common mental illness, affects more than 300 million people worldwide and has serious health outcomes^51^. Considering the adverse drug reactions to antidepressants, aromatic drugs via inhalation provide a convenient and effective treatment option for many patients to relieve their symptoms. Moreover, herbs provide an excellent source for the extraction and development of drugs for depression, including some aromatic drugs and volatile oils, such as LIG. LIG is the main volatile oil in angelica sinensis, which has a definite effect on brain disorders.

In this study, we first obtained 150 possible targets of LIG for an anti-depressive effect by a network pharmacology approach. We constructed the PPI network for these targets and identified the core targets, including MAPK3, EGF, MAPK14, CCND1, IL6, CASP3, IL2, MYC, TLR4, AKT1, ESR1, TP53, HIF1A, SRC, STAT3, AR, IL1B, and CREBBP. GO analysis showed that many biological processes, including protein serine/threonine kinase activity, regulation of DNA-binding transcription factor activity, and epithelial cell proliferation, were associated with the anti-depressive effects of LIG. The serine/threonine kinase is encoded by the AKT1 gene and can be activated by extracellular signals through a PI3K-dependent mechanism. KEGG analysis of core targets confirmed a significant association with PI3K/AKT signaling pathway, MAPK signaling pathway, etc. Molecular docking of LIG with core targets showed strong affinities of LIG to AKT1, MAPK14, and ESR1. Finally, we further validated the interactions between the proteins and LIG by MD simulations.

The mechanisms of depression are complex, but the PI3K/AKT signaling pathway is significantly associated with anti-depressive effects. A study found that acute systemic trefoil factor 3 administration increased the levels of phosphorylated AKT in the basolateral amygdala, which exerts an anti-depressive effect^52^. PI3K/AKT signaling is associated with the etiology of depression, and antidepressants that act on 5-HT neurotransmission and lithium can activate AKT^53^. For example, the antidepressant fluoxetine inhibits 5-hydroxytryptamine reuptake in the central nervous system^54^ and can improve neuronal survival, associated with its upregulation of phosphorylated AKT protein expression^55^. Therefore, the PI3K/AKT signaling pathway is crucial in treating depression. Our molecular docking results showed that LIG had the strongest affinity to AKT1 (− 7.8 kcal/mol), much smaller than − 1.2 kcal/mol. The amino acid residues bound to AKT1 by LIG include ASN-204 and SER-205. AKT is a serine/threonine kinase that can be activated by catalyzing the phosphorylation of its own serine and threonine sites^56^. Therefore, we hypothesized that the interaction of LIG with SER-205 could activate AKT1 activity. Indeed, several brain studies have provided some supporting evidence. For example, LIG attenuates cerebral infarction volume, nerve injury, and hippocampal neuronal damage by activating the PI3K/AKT pathway^57^. And Z-LIG, an isomer of LIG, exhibited an anti-depressive effect in a rat depression model^14^. The redocking suggested that the RMSD values of the native ligands were less than 2 Å, which indicated that our molecular docking scheme was reliable. Also, the MD simulation of AKT1-LIG showed that the average RMSD of AKT1 was 0.23 nm and the average RMSD of LIG was 0.09 nm, indicating the complex system's stability. The RMSF result indicated that AKT1 had high flexibility in the 6200–6300 atomic region, which may be important for the protein to perform its function. In addition, the MD result suggested that based on an angle ≤ 30° and a radius ≤ 0.35 nm, AKT1 and LIG could form up to 2 hydrogen bonds, and the non-bonded interaction between AKT1 and LIG was − 131.3 ± 3.8 kJ/mol, which provides evidence for the interaction of both.

The MAPK signaling pathway can integrate external signals and produce biological effects^58^. In addition, MAPK14 responds to multiple extracellular stimuli in the brain^59^ and is involved in synaptic function and dysfunction^60^. Studies have also confirmed that MAPK14 may play an important role in depression. For example, N-acetylcysteine can inhibit MAPK14-related signaling in depressed rats to attenuate neuronal damage^61^, and inhibition of the MAPK14 signaling pathway can exert antidepressant-like effects^62^. Our molecular docking suggested that LIG had an excellent affinity at the inhibitor binding site of MAPK14. The RMSD values of both MAPK14 and LIG in MD simulations were below 0.3 nm, which indicated the stability of the MAPK14-LIG complex. In addition, hydrogen bond monitoring reported the formation of hydrogen bonds between MAPK14 and LIG, and their non-bonded interaction energy was − 136.6 ± 6.9 kJ/mol. In addition, molecular docking also revealed that LIG might interact with ESR1. Although hydrogen bond monitoring in MD simulations did not yield results consistent with molecular docking, the non-bonded interaction energy of ESR1 and LIG suggested a possible mode of action for both. Estrogen can regulate neurotransmitter turnover and 5-hydroxytryptamine receptor function through ESR1^63^. Several studies have confirmed the association of ESR1 with postpartum depression^64–66^. Also, the antidepressant clomipramine can alter the expression of estrogen receptors in the brain regions of male adult rats^67^. Therefore, MAPK14 and ESR1 may be other critical targets for LIG to exert an anti-depressive effect.

**Figure 7:**
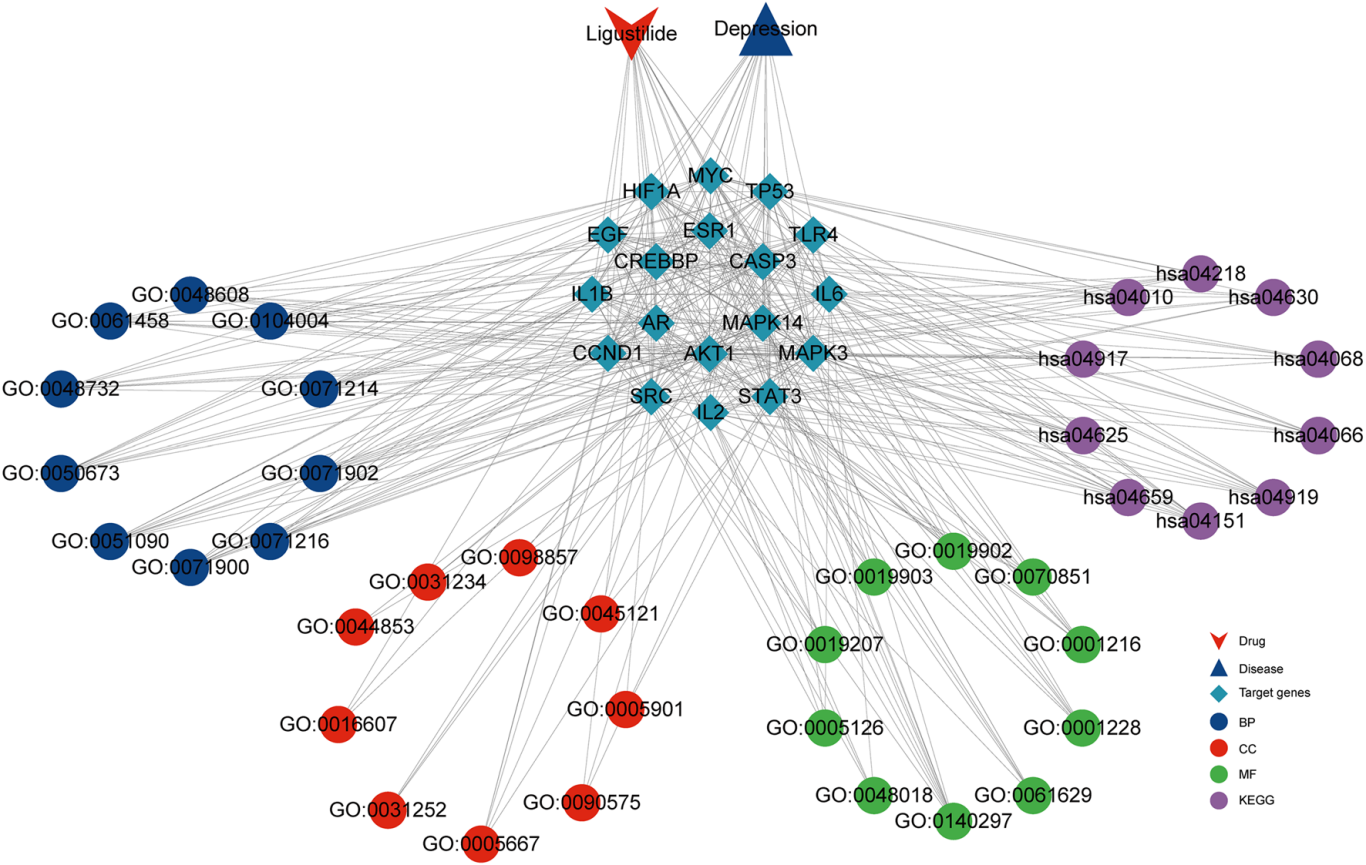
The network of LIG anti-depressive targets and the interactions of related pathways. *LIG* ligustilide, *BP* biological process, *CC* cellular component, *MF* molecular function, *GO* Gene Ontology, *KEGG* Kyoto Encyclopedia of Genes and Genomes.

In summary, this is the first application of network pharmacology, molecular docking, and MD simulation to systematically explore the potential targets and mechanisms of the anti-depressive effects of LIG. We found that LIG may exert an anti-depressive effect through binding to targets such as AKT1, MAPK14, and ESR1 (Fig. 7). However, it should be noted that our study has some limitations. First, the reliability and accuracy of the predictions in this study depend on the quality of the database data. Second, the specific mechanism of the anti-depressive effect of LIG still needs to be verified by animal experiments or even clinical trials.

## Conclusions

Based on network pharmacology, molecular docking, and molecular dynamics simulations, this study found that LIG exerted an anti-depressive effect through multiple targets, including AKT1, MAPK14, and ESR1, and the pathways of PI3K/AKT and MAPK. Our study provides a new strategy to explore the molecular mechanisms of LIG in treating depression.

## Supplementary Information

Supplementary Table S1.

Supplementary Table S2.

Supplementary Table S3.

Supplementary Table S4.

Supplementary Table S5.

## Data Availability

The datasets generated and/or analysed during the current study are available in the figshare repository, 10.6084/m9.figshare.21063736.v1.
